# Both Castration and Goserelin Acetate Ameliorate Myocardial Ischemia Reperfusion Injury and Apoptosis in Male Rats

**DOI:** 10.1155/2014/206951

**Published:** 2014-03-04

**Authors:** Najah R. Hadi, Fadhil G. Yusif, Maitham Yousif, Karrar K. Jaen

**Affiliations:** ^1^Department of Pharmacology and Therapeutics, College of Medicine, Kufa University, P.O. Box 83, Kufa, Iraq; ^2^Department of Surgery, College of Medicine, Kufa University, Kufa, Iraq; ^3^College of Science, Al-Qadisiya University, Al Diwaniyah, Qadisiyyah Province, Iraq

## Abstract

Although reperfusion of an ischemic organ is essential to prevent irreversible tissue damage, it may amplify tissue injury. This study investigates the role of endogenous testosterone in myocardial ischemia reperfusion and apoptosis in male rats. *Material and method*. Twenty four male rats were randomized into 4 equal groups: Group (1), sham group, rats underwent the same anesthetic and surgical procedure as the control group except for LAD ligation; Group (2), Active control group, rats underwent LAD ligation; Group (3), castrated, rats underwent surgical castration, left 3wks for recovery, and then underwent LAD ligation; and Group (4), Goserelin acetate treated, rats received 3.6 mg of Goserelin 3 wks before surgery and then underwent LAD ligation. At the end of experiment, plasma cTn I, cardiac TNF-**α**, IL1-**β**, ICAM-1, and Apoptosis level were measured and histological examination was made. *Results*. Compared to sham group, the levels of myocardial TNF-**α**, IL-1**β**, ICAM-1, apoptosis, and plasma cTn I were significantly increased (*P* < 0.05) in control group and all rats showed significant myocardial injury (*P* < 0.05). Castration and Goserelin acetates significantly counteract the increase in myocardial levels of TNF-**α**, IL-1**β**, ICAM-1, plasma cTn I, and apoptosis (*P* < 0.05) and significantly reduce (*P* < 0.05) the severity of myocardial injury. We conclude that castration and Goserelin acetates ameliorate myocardial I/R injury and apoptosis in rats via interfering with inflammatory reactions.

## 1. Introduction

Ischemic heart disease is one of the major leading causes of death for both men and women. Depriving the organ from its blood supply has long been documented as a critical factor in the clinical outcome of stroke, hemorrhagic shock, myocardial infarction, and organ transplantation. Although the restoration of blood flow to an ischemic organ is essential to prevent permanent tissue damage, reperfusion may increase tissue injury in excess of that produced by ischemia alone. Restoration of blood flow to ischemic myocardium results in the ischemia reperfusion (I/R) injury [1].

Cellular damage after reperfusion of previously viable ischemic tissue is defined as ischemia reperfusion (I/R) injury [2]. I/R injury may occur in a variety of clinical situations, including reperfusion after thrombolytic therapy, coronary angioplasty, organ transplantation, and cardiopulmonary bypass. Reperfusion of ischemic tissue results in both a local and systemic inflammatory responses that, in turn, may give rise to wide spread microvascular dysfunction and changed tissue barrier and function. If sever enough, the inflammatory response after I/R may even lead to systemic inflammatory response or multiple organ dysfunction syndrome, which account for up to 30–40% of intensive care unit mortality [3].

Gender differences have been distinguished in I/R injury [4–10], with several studies connecting the sex hormone estrogen in the cardioprotection found in females [11–15]. In contrast, testosterone has received little attention. Presently the majority of evidence points toward the detrimental effects of testosterone on myocardium, possibly by adverse effects on lipoproteins, thrombosis, and cardiac hypertrophy [16–19]. Indeed, some studies established that chronic endogenous testosterone has a deleterious effect in the isolated rat heart subjected to I/R [20].

Recent advances in our understanding of cell death during ischemia reperfusion implicate two forms of cell death in the pathology of myocardial infarction that is to say necrosis and apoptosis [21]. Apoptosis cell death in rat heart has been established to be induced by prolonged episode of ischemia alone in absence of reperfusion [22, 23]. Some studies have suggested that reperfusion accelerates the apoptotic cell death process initiated during ischemia [22, 24–26]. In contrast, several studies suggest that the apoptotic component of cell death is triggered at time of reperfusion and does not manifest during the ischemic period [27].

Therefore, evidence suggests that the apoptotic component of cell death is either generated or accelerated during the reperfusion phase. The fact that the apoptosis is an energy dependent process and ATP levels are depleted during ischemia and replenished on reperfusion may explain why the apoptotic component of cell death is associated with reperfusion [28].

## 2. Material and Method

### 2.1. Preparation of Animals

A total number of 24 adult male albino rats weighting (200–250 g) were purchased from Animal Resource Center, National Center for Drug Control and Research. They were housed in the animal house of Kufa university/College of Medicine in a temperature-controlled (25° ± 1 C) room (humidity was kept at 60–65%) with alternating 12-hr light/12-hr dark cycles and were allowed free access to water and chow diet until the start of experiment.

### 2.2. Study Design

After the 1st week of acclimatization, the rats were randomized into 4 groups (6 rats in each group) as follow.

1. In sham group, rats underwent the same anesthetic and surgical procedures (for an identical period of time for regional myocardial ischemia and reperfusion) but without LAD ligation.
2. In control group (induced untreated), rats underwent surgical operation for LAD ligation and were subjected to 25 min of ischemia and 40 min of reperfusion.
3. In castrated group, surgically castrated rats were left 4wks for recovery [20] and then underwent surgical LAD ligation and were subjected to 25 min of ischemia and 40 min of reperfusion.
4. In treated group (Goserelin treated), rats of this group take a S.C injection of Goserelin acetate (LHRH analogue) 3 wks before the surgery [29] and then were subjected to surgical LAD ligation with 25 min ischemia and 40 min of reperfusion.

### 2.3. Surgical LAD Ligation

The procedure of LAD ligation in rats is modified from that mentioned in previous study with modification [30]. The rats were anesthetized by intraperitoneal (IP) injection of 100 mg/kg ketamine and 5 mg/kg xylazine [31]. when the rat anesthetized (within 5–10 min), we placed it in supine position, then, fix the four limbs, tail and extended the head. The trachea is intubated with a cannula sized either 22 fg or 20 fg according to the weight of animal as the small catheter is reserved for the smaller animal. And the tube is connected tightly to the ventilation machine. With scissors and fine pickup, (2-3) cm long skin incision was made over the left thorax area starting from midline, just 30 mm above the xiphoid process to the left anterior axillary fold. The dissection continues until reaching the pleura and exposes the heart. Zoom in the microscope on the heart and then the pericardium is opened using two pairs of small micropickup. The left ventricle (LV) with its partly overlying left auricle (atrium) is now visible. The left anterior descending coronary artery (LAD) is ligated just distal to the left auricle, the distance from the ligation site to the left auricle being the length of both tips of a pair of rounded forceps as this is equal to (2-3) mm. The LAD is ligated with an 8 zero Prolene suture. The ligation continues for 25 min; then the ligation is terminated by cutting the ligature by using microscissor. At this time the reperfusion time is started to be calculated, then closing the chest wall by enclosing the ribs with figure eight of 4 zero silk suture. The pectoral muscles should be moisturized and returned back into the original position (first the minor and then the major partly overlying it); after that, the skin is closed with 4 zero silk suture, watching for the spontaneous breathing, and when it is sufficient, then the decision is made for gentle and careful extubation after freeing the mouse from tapes. Finally, the mouse should be transferred into clean cage oxygenated with 100% oxygen and placed near fair heating lamp. The reperfusion time is calculated from the moment of removing the ligature and the reperfusion is continued for 40 min. At the end of reperfusion period, the animal is reanesthetized and placed in the supine position with its limbs immobilized, the chest reopened, and the heart exposed, and then a needle of the syringe is introduced into right ventricle to aspirate around (5–8) mL of blood for later blood analysis. After that, the heart is cut from the great vessels and mediastinum. The atria are removed.

### 2.4. Blood Sampling for Measurement of Plasma cTn I

At the end of experiment, about 2 mL of blood was collected from the heart. The blood sample was placed in a tube containing disodium EDTA (22 mg/mL) as anticoagulant and mixed thoroughly and then centrifuged at 3000 rpm for 15 min. Then it was used for determination of plasma cTn I By ELISA with a commercially available ELISA kit (Literature of kit by life Diagnostic, USA) according to the manufacturer's instructions.

### 2.5. Myocardial TNF-*α*, IL-1*β*, and ICAM-1 Measurement

The excised heart tissues were rinsed with ice cold saline to remove any red blood cells or clots and then homogenized with a high intensity ultrasonic liquid processor in 1 : 10 (w/v) phosphate buffered saline that contained 1% Triton X-100 and a protease inhibitor cocktail [32]. The homogenate was centrifuged at 2,500 g for 20 min at 4°C. The supernatant was collected for determination of TNF-*α*, IL-1*β*, and ICAM-1 by ELISA with a commercially available ELISA kit (Literature of kit by life Diagnostic, USA) according to the manufacturer's instructions.

### 2.6. Tissue Sampling for Histopathology

The cardiac sections for histopathological study were fixed in 10% formalin and embedded in paraffin [33]. The sections were stained with hematoxylin and eosin (H&E) after fixation. Evaluate scores were performed by an investigator who was blinded to the experimental treatment groups. The following morphological criteria [34] were used to assess the histopathological damage: score 0, no damage; score 1 (mild), interstitial edema and focal necrosis; score 2 (moderate), diffuse myocardial cell swelling and necrosis; score 3 (severe), necrosis with the presence of contraction bands, neutrophil infiltration, and the capillaries were compressed; and score 4 (highly severe), widespread necrosis with the presence of contraction bands, neutrophil infiltration, capillaries compressing, and hemorrhage.

### 2.7. Myocardial Apoptosis Level Measurement

1. The part of ventricular samples that were used for measurement of apoptosis level underwent lyses by Trypsin. Trypsin is a serine protease commonly used for detachment of adherent cell lines and dissociation of tissues. The concentration of trypsin can range from 0.025% to 0.5%. Incubating cells with too high trypsin concentration for too long time period will damage cell membranes and kill the cells. For the dissociation of tissues, trypsin has been used alone or as a supplement to other enzymes.
2. Transfer 1,000 to 10,000 nonfixed cells to each well.
3. Centrifuge plate at 200 g for 5 minutes. Remove medium, add 200 *μ*L of fixative (80% methanol in PBS), and incubate at room temperature for 30 minutes. Then the measurement is done by ELISA with a commercially available ELISA kit (ssDNA Apoptosis ELISA Kit, CHEMICON International, Inc. USA).

### 2.8. Presentation of Data and Statistical Analysis

Data were expressed as mean ± SEM. Quantitative variables were tested for statistical significance of difference between more than 2 groups by One-Way ANOVA test followed by Post Hoc. LSD test for multiple comparisons. Nonparametric tests were used to assess the statistical significance of histopathological parameter. Myocardial lesions are a nonnormally distributed variable. The statistical significance of difference between more than 2 groups was assessed by Kruskal-Wallis test, while Mann-Whitney *U* test was used for the difference between 2 groups. In all tests, *P* < 0.05 was considered to be statistically significant.

## 3. Results

### 3.1. Myocardial TNF-*α*, IL-1*β*, and ICAM-1

**Figure 1:**
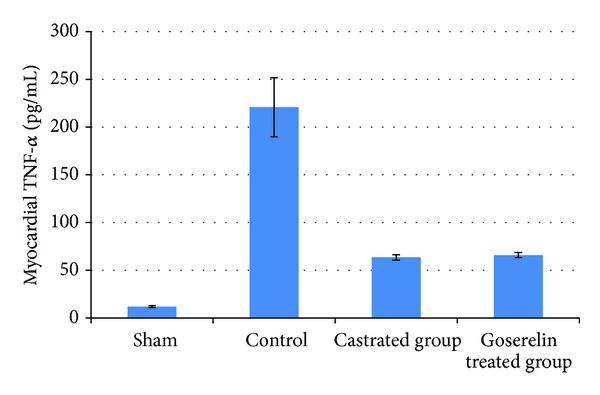
The mean of myocardial TNF-*α* level (pg/mL) in the four experimental groups at the end of the experiment.

**Figure 2:**
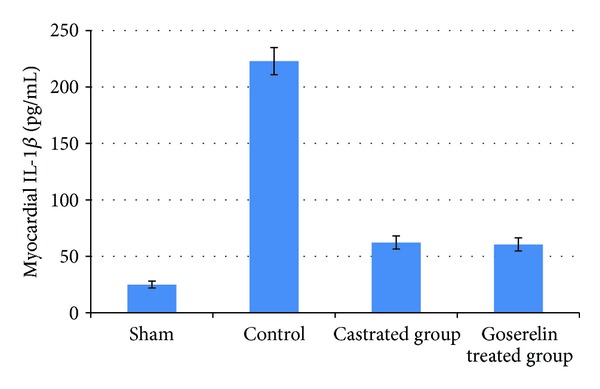
The mean of myocardial IL-1*β* level (pg/mL) in the four experimental groups at the end of the experiment.

**Figure 3:**
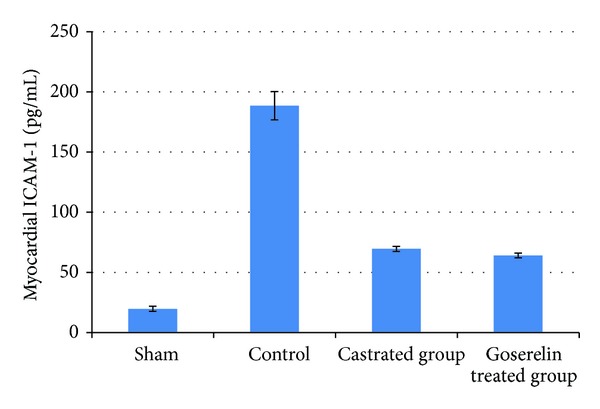
The mean of myocardial ICAM-1 level (pg/mL) in the four experimental groups at the end of the experiment.

Compared with the sham group, levels of myocardial TNF-*α*, IL-1*β*, and ICAM-1 were increased (*P* < 0.05) in control group. Both castration and Goserelin acetate injection significantly counteract the increase in myocardial levels of TNF-*α*, IL-1*β*, and ICAM-1 (*P* < 0.05), but there was no significant difference between castrated group and Goserelin treated group as shown in Figures 1, 2, and 3.

### 3.2. Plasma cTn I Level

**Figure 4:**
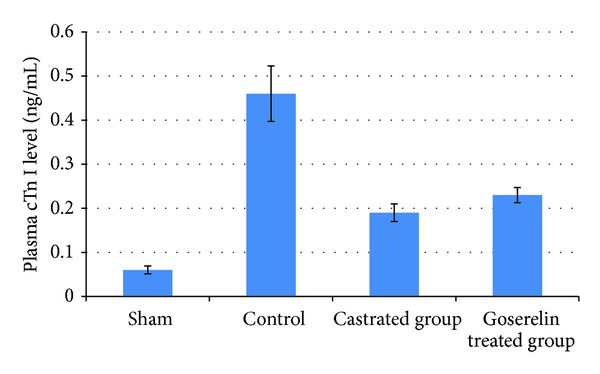
The mean of plasma cTn I level (ng/mL) in the four experimental groups at the end of the experiment.

Compared with the sham group, levels of plasma cTn I were increased (*P* < 0.05) in control group. Both surgical castration and Goserelin injection significantly counteract the increase in level of plasma cTn I (*P* < 0.05), but there was no significant difference between castrated group and Goserelin treated group as shown in Figure 4.

### 3.3. Myocardial Apoptosis Level

**Figure 5:**
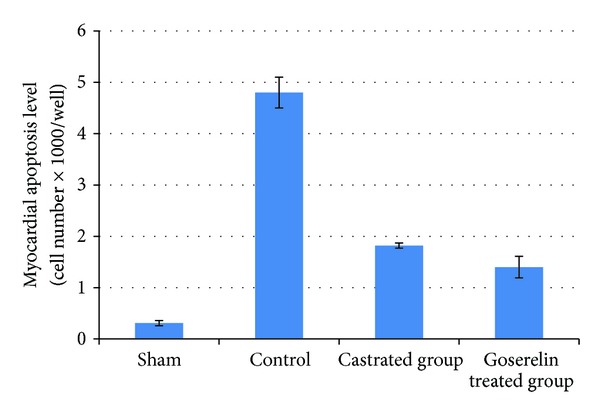
The means of myocardial apoptosis level in the four experimental groups at the end of the experiment.

Compared with the sham group, myocardial apoptosis levels were increased (*P* < 0.05) in control group. Both surgical castration and Goserelin injection significantly counteract the increase in myocardial apoptosis level (*P* < 0.05), but there was no significant difference between castrated group and Goserelin treated group as shown in Figure 5.

### 3.4. Histopathological Findings

**Figure 6:**
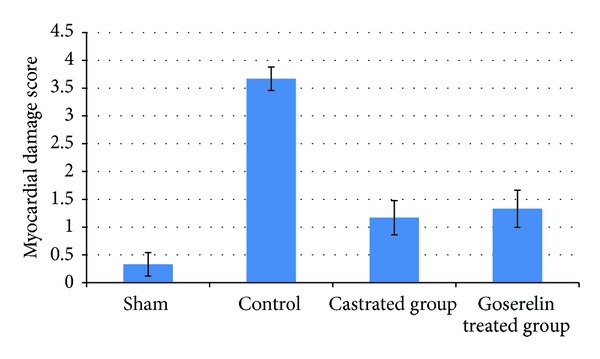
The means of myocardial damage score in the four experimental groups at the end of the experiment.

**Figure 7:**
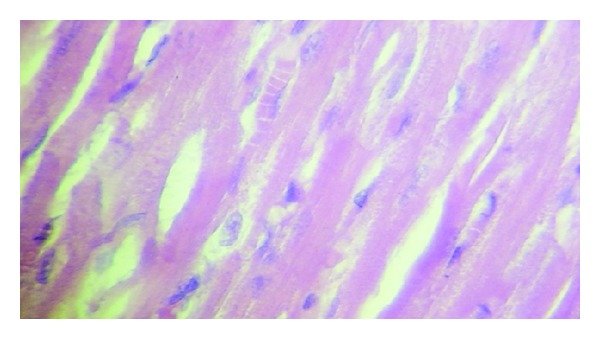
Photomicrograph of section of normal rats myocardium shows the normal architecture. The section stained with H&E (×40).

**Figure 8:**
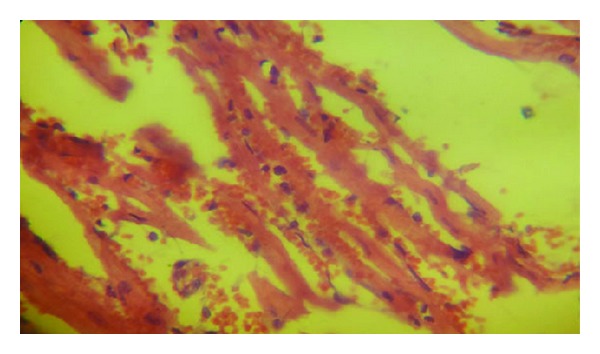
Photomicrograph of heart section with highly sever injury, showing extensive hemorrhage and contraction band. The section stained with H&E (×40).

**Figure 9:**
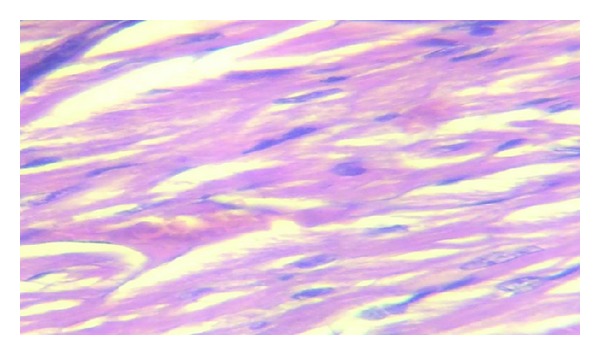
Photomicrograph of heart section of castrated rat, showing mild injury. The section stained with H&E (×40).

**Figure 10:**
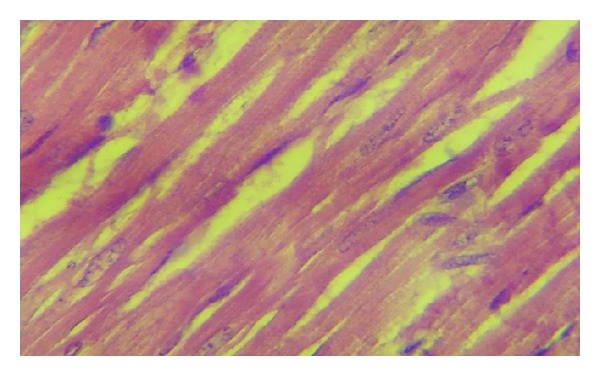
Photomicrograph of heart section treated with Goserelin, showing mild injury. The section stained with H&E (×40).

Histologically, all control group rats showed a significant myocardial injury (*P* < 0.05) compared with sham group as shown in Figures 6, 7, and 8. Both castration and Goserelin injection markedly reduced (*P* < 0.05) the severity of heart injury in the rats which underwent the regional ischemia-reperfusion procedure, but there was no significant difference between castrated group and Goserelin treated group as shown in Figures 6, 8, 9, and 10.

## 4. Discussion

The main findings of the present study were that after myocardial I/R, compared with control group, both castrated group and Goserelin treated group (1) exhibited a lower histopathological damage, (2) had decreased proinflammatory cytokine production (TNF-*α*, IL-1*β*, and ICAM-1), (3) exhibited a lower apoptotic injury, and (4) there were no significant differences between castrated group and Goserelin treated group. The role of testosterone in cardiac injury may be very important because the heart can accumulate testosterone at higher concentrations than other androgen target organs [35] and functional androgen receptors are found in isolated cardiac myocytes [36]. Role of testosterone in cardiac injury is controversial; some studies indicated that testosterone was reported to confirm cardioprotection against ischemia reperfusion by upregulating the cardiac alpha(1)-adrenoceptor and enhancing the effects of stimulation of this adrenoceptor [37]. On the contrary, other studies hypothesized that testosterone may exert deleterious effects on myocardial proinflammatory cytokine production through proinflammatory and/or the proapoptotic properties of endogenous testosterone in ischemia reperfusion [20]. Furthermore, exogenous testosterone supplementation increased apoptosis in adult rat ventricular myocytes during I/R injury [38].

The relationship between apoptotic and necrotic cell death during ischemia reperfusion injury is unresolved with some authors showing that there may be significant overlap in terms of early signaling steps between these two pathways, this observation may be useful in development of future therapeutic targets for clinical use. Zhao et al. have used a canine model of ischemia reperfusion injury to demonstrate the contribution of necrotic and apoptotic cell death. They established that both forms of cell death occur together during the reperfusion phase with necrotic cell death peaking after 24 hr. of reperfusion and apoptotic cell death increasing up to 72 hr. of reperfusion [39]. Other studies have showed that the pharmacological inhibition of the apoptotic signaling cascade during the reperfusion phase is able to attenuate both the apoptotic and the necrotic cell death [40–43]. They suggested that the apoptotic death can evolve into necrotic cell death. To the best of our knowledge, there is no previous study that used Goserelin acetate to produce a chemical castration for male rats but there are many studies that used the testosterone receptor blockers as flutamide to block the testosterone receptors. Wang et al. demonstrated that male rats of both (1) treated group with testosterone receptor blocker (flutamide) and other groups (2) with surgical castration when were subjected to I/R show a decrease in production of proinflammatory cytokines (TNF-*α*, IL1*β*, and ICAM-1) compared with untreated control group [20], explaining the possible role of testosterone in promoting the myocardial inflammatory response. Other studies report protected cardiac performance in animals with testosterone depletion or testosterone receptor blockade after trauma and hemorrhage and suggest that endogenous testosterone may have a negative effect on the heart subjected to I/R.

Wang et al. demonstrated that both male rats treated with testosterone receptor blocker (flutamide) and male rats underwent surgical castration when subjected to I/R show a decrease in the activation of proapoptotic signaling cascade (caspase-3, caspase-11) and increase antiapoptotic BCL2 compared with control untreated group so the net result is decrease in apoptosis [20]. Crisostomo et al. showed that female rat hearts had better functional recovery after acute ischemia than male rat and expressed less myocardial TNF-*α*, IL1-*β* (mRNA and protein) than male hearts subjected to the same I/R insult [44]. The present study showed that there was a significant lowering (*P* < 0.05) in plasma level of cTnI in both castrated group and Goserelin group compared to control group. Crisostomo et al. also demonstrated that in rat hearts devoid from chronic exposure to testosterone, a single dose of exogenous testosterone increases apoptosis level [44].

Findings in the present study according to apoptosis level are in agreement with the above 2 studies.

The present study showed a marked decrease in ischemic injury in both castrated and Goserelin treated groups (*P* < 0.05) compared with the control group but there was no significant difference between castrated group and Goserelin treated group. 50% of castrated group show mild cardiac damage (interstitial edema and focal necrosis), 33.33% of castrated group show moderate cardiac damage (diffuse myocardial cell swelling and necrosis), and 16.67% of castrated group show normal tissue appearance. Cavasin et al. showed that castrated male rats when subjected to the ischemia have significantly decreased in the severity of histological finding of ischemia so that there are less ischemic expansion and less neutrophil infiltration at the infarction border than the noncastrated male control group [45]. Histopathological finding in Goserelin treated group explains that 33.33% of treated group show mild myocardial damage (interstitial edema with focal necrosis), 50% of treated group show moderate myocardial damage (diffuse myocardial cell swelling and necrosis), and 16.67% of treated group show normal myocardial appearance. Regarding Goserelin treated group, to the best of our knowledge there is no study yet available to compare our data.

This study is further supporting the role of inflammatory cytokine (IL-1*β*, TNF-*α*, and ICAM-1) in the pathology of regional myocardial I/R and myocardial apoptosis during regional I/R injury.

Both surgical castration and Goserelin administration reduce the level of inflammatory mediators in myocardial tissues, which may provide mechanistic answer for their protective effect. Both surgical castration and Goserelin administration ameliorate myocardial injury and apoptosis in regional I/R induced by LAD ligation in rats as evidenced by reduction in the release of cardiac specific enzyme troponin I.

The findings of the present study support the idea that endogenous testosterone may have deleterious effect on myocardial injury and apoptosis during regional I/R.
